# High accuracy EEG signal classification for brain computer interfaces using advanced neural architectures

**DOI:** 10.3389/fnins.2026.1752176

**Published:** 2026-02-18

**Authors:** Daicheng Lin, Qi Zhang, Huan Chen, Yanjie Lu, Haiting Chen, Lianfeng Li, Abdulilah Mohammad Mayet, Guodao Zhang, Xinjun Miao, Xianke Qiu

**Affiliations:** 1 Department of Emergency, Wenzhou Central Hospital, Wenzhou, Zhejiang, China; 2 Wenzhou Central Hospital, Affiliated to Wenzhou Medical University, Zhejiang, China; 3 Institute of Intelligent Media Computing, Hangzhou Dianzi University, Hangzhou, China; 4 Shangyu Institute of Science and Engineering Co. Ltd., Hangzhou Dianzi University, Shaoxing, China; 5 School of Data Science and Artificial Intelligence, Wenzhou University of Technology, Wenzhou, China; 6 Network Information Center, The Maternal and Child Health Hospital of Guangxi Zhuang Autonomous Region, Nanning, China; 7 Electrical Engineering Department, King Khalid University, Abha, Saudi Arabia; 8 Fujian Key Laboratory of Big Data Application and Intellectualization for Tea Industry, Wuyi University, Wuyishan, China

**Keywords:** brain-computer interface, EEG, feature extraction, GMDH neural networks, motor movement classification

## Abstract

**Introduction:**

This study proposes advanced neural network architectures for classifying specific motor-related electroencephalography (EEG) tasks, employing deep feature extraction techniques. We analyzed EEG data from the MILimbEEG dataset, consisting of recordings from 60 individuals as they performed eight distinct motor movements: baseline with eyes open, left-hand closing, right-hand closing, dorsiflexion and plantarflexion of both the left and right feet, as well as rest periods between tasks. The high precision achieved in this study underscores the efficacy of sophisticated computational models like the GMDH network in enhancing the interpretation of EEG signals for the development of brain-computer interfaces (BCIs). This research significantly advances the potential of EEG as a reliable modality for BCIs, effectively translating brain activity into actionable commands suitable for neurorehabilitation and assistive technologies.

**Methods:**

For each of the 16 electrodes used in the recordings, 10 critical features were extracted, resulting in a comprehensive set of 160 features per sample that encapsulate the intricate brain activities associated with each task. A Group Method of Data Handling (GMDH) neural network, structured with eight hidden layers and a decremental arrangement of neurons from 40 in the first to 5 in the last, was utilized to classify these tasks.

**Results:**

This network configuration achieved an impressive classification accuracy of approximately 96%, demonstrating a robust capability to accurately decode EEG signals tied to specific motor actions.

**Discussion:**

The high precision achieved in this study underscores the efficacy of sophisticated computational models like the GMDH network in enhancing the interpretation of EEG signals for the development of brain-computer interfaces (BCIs). This research significantly advances the potential of EEG as a reliable modality for BCIs, effectively translating brain activity into actionable commands suitable for neurorehabilitation and assistive technologies. Our findings contribute substantially to the BCI field, promising to improve clinical outcomes by enabling more precise and effective interaction with neurorehabilitation devices.

## 1 Introduction

Electroencephalography (EEG) is a non-invasive technique that records electrical activity of the brain via electrodes placed on the scalp. The ability to monitor and interpret these signals is critical for a wide range of applications, from clinical diagnostics and neurotherapy to advanced interfaces between humans and machines. EEG signals are inherently complex, reflecting the dynamic interplay of neural circuits involved in sensory processing, cognitive functions, and motor responses. Classifying these signals accurately opens up profound possibilities for understanding brain functions, diagnosing neurological disorders, and developing brain-computer interfaces (BCIs) that can translate thoughts into actions. As such, the classification of EEG signals using advanced computational models not only enhances our ability to interpret neural signals—specifically identifying patterns associated with motor or cognitive activities—but also paves the way for innovative therapeutic strategies and assistive technologies that can significantly improve quality of life for individuals with impaired cognitive or motor functions.

Machine Learning (ML) approaches based on Deep Neural Networks (DNN) are celebrated for significantly boosting the reliability and precision of task detection in EEG-driven systems for prosthetic limbs (Al-Haddad and Jaber, 2023). Biomedical engineers are increasingly investigating Artificial Intelligence (AI) techniques that employ EEG data for diagnostic and classification tasks (Tuncer and Bolat, 2022; Li et al., 2023; Ali et al., 2023). Promising results have been observed with deep learning algorithms focused on emotion recognition using EEG, which benefit from advanced feature extraction techniques (Li et al., 2023). Additionally, the accuracy in representing and classifying EEG signals has been enhanced by applying multi-task learning algorithms (Choo et al., 2023). In the area of EEG Motor Imagery (MI), Convolutional Neural Networks (CNNs) have proven effective, particularly those that utilize several layers to analyze spatial and temporal data (Amin et al., 2019). Meanwhile, the complex legal requirements for medical devices have been scrutinized, highlighting the need for innovative, data-driven methods in their design and approval (Arnould et al., 2021). Moreover, the development of prosthetic sockets, traditionally dependent on expert analysis, has recently started integrating machine learning methods (Dickinson et al., 2021). These studies collectively highlight the crucial role of AI in tackling complex issues within various branches of biomedical engineering.

In prosthetic limb control systems, there has been a traditional reliance on electromyography (EMG) data (Baygin et al., 2022; Tuncer et al., 2020). Various approaches for analyzing hand movements and activities using surface EMG data have been established (Fatimah et al., 2021; Karnam et al., 2022). Initial investigations have explored gesture detection control in bionic hands through the use of surface EMG data (Shi et al., 2018). While EMG-based systems are highly effective, integrating EEG represents a notable improvement. Employing EEG to monitor brain activity provides a more intuitive and potentially more accurate control system compared to EMG, which records muscle electrical activities. Recent research has shown promise for superior control systems utilizing a hybrid EEG-FNIRS Brain-Computer Interface that engages ensemble learning and nonlinear feature extraction techniques (Maher et al., 2023).

In the realm of biomedical engineering, there is a significant shift toward more sophisticated computational techniques, particularly within the domain of prosthetic technology. Noteworthy progress has been observed in Brain-Computer Interface (BCI) systems that integrate machine learning algorithms on Field Programmable Gate Arrays (FPGA), notably in accurately determining motor intentions for prosthetic hand movements (Constantine et al., 2021). Studies focusing on cost-effective, high-performance prosthetics for the upper limbs have utilized EEG data alongside cutting-edge deep learning methods, like LSTM networks that are refined with Genetic Algorithms (GA) to classify motion intentions in real time (Kansal et al., 2023). Additionally, haptic feedback mechanisms that mimic the activities of mechanoreceptors to provide tactile feedback have been incorporated into EEG-based control systems for economical robotic hand prostheses, enhancing the capability to recognize objects and shapes (Cutipa-Puma et al., 2023).

In recent years, the application of deep convolutional neural networks (CNNs) in brain-computer interface (BCI) research has led to significant advancements in the classification of EEG signals. These models, known for their ability to capture complex spatial and temporal patterns, have achieved remarkable performance in various domains. For instance, a study on EEG-based mobile robot control demonstrated the effectiveness of a CNN integrated with a robotic operating system (ROS) for enhancing real-time control. While this approach showed high accuracy in translating EEG signals into actionable commands, its computational intensity posed challenges for offline applications and resource-constrained settings (Ghinoiu et al., 2024). Similarly, a hybrid deep learning framework combining CNNs with autoencoders was developed for EEG-based emotion recognition. This ensemble model exhibited robust accuracy in detecting emotions but required significant computational resources, limiting its adaptability for portable systems. The reliance on high-performance hardware emphasized the gap in implementing such models for real-time applications (Yousefipour et al., 2024). Another noteworthy example is a CNN-based architecture tailored for emotion recognition in a continuous valence-arousal-dominance (VAD) space. By employing 2D convolutional layers, this model effectively captured the spatial and temporal dynamics of EEG signals. However, the computational demands of the system highlighted its limitations in being deployed in environments with limited hardware capabilities (Jon et al., 2024). In the realm of motor imagery EEG classification, a novel CNN architecture using a sliding window technique achieved state-of-the-art performance in distinguishing motor tasks. Despite its efficacy, the high computational overhead raised concerns regarding its feasibility for real-time or offline BCI applications (Singh et al., 2024). Additionally, research utilizing a ResNet-101 architecture for improved motor imagery classification highlighted the model’s ability to extract nuanced features. Nevertheless, its deep structure led to significant resource consumption, making it challenging for integration into practical systems (Sharma et al., 2024). These studies collectively underscore the potential of CNN-based approaches in advancing EEG signal processing. However, they also reveal a critical limitation: the computational complexity inherent in deep architectures, which necessitates powerful and often costly hardware. This constraint poses significant challenges for real-time, offline, or embedded systems, particularly in neurorehabilitation and assistive technologies.

Although the individual components of the proposed framework—manual EEG feature extraction and GMDH-based learning—have been explored in prior studies, the originality of this work lies in their task-specific integration and systematic design for multi-class motor task decoding under computational constraints. First, this study addresses an eight-class EEG motor task classification problem involving both upper- and lower-limb movements, which is more challenging than the binary or four-class motor imagery tasks commonly investigated in lightweight BCI literature. Second, we propose a decremental multi-layer GMDH architecture specifically tailored to EEG-derived features, enabling progressive feature abstraction while maintaining strict control over model complexity. This architectural design has not been systematically explored in prior EEG-based GMDH applications. Third, by combining physiologically interpretable time- and frequency-domain EEG features with a self-organizing polynomial network, the proposed approach bridges traditional feature engineering and adaptive learning. This integration yields a robust and computationally efficient alternative to both classical shallow classifiers and data-hungry deep learning models. Finally, the proposed framework is quantitatively validated in terms of both classification performance and computational efficiency, demonstrating that high accuracy can be achieved without relying on deep architectures. These aspects collectively distinguish the present work from existing lightweight EEG classification methods.

**Figure 1:**
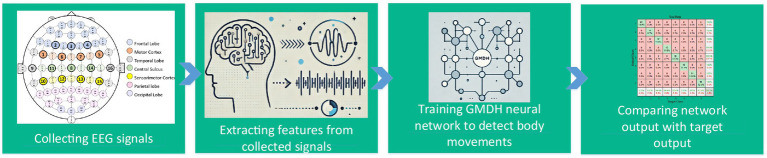
Flowchart of the proposed methodology, illustrating the sequential steps of data collection, feature extraction, GMDH neural network implementation, and output evaluation.

Figure 1 provides a flowchart summarizing the key steps of the proposed methodology. The process begins with the collection of EEG signals during predefined motor tasks, followed by the extraction of significant features that encapsulate the temporal and spectral characteristics of the signals. These features serve as inputs to the GMDH neural network, designed with a decremental neuron architecture in its hidden layers to optimize classification accuracy. Finally, the outputs of the network are compared with the target outputs to evaluate performance, ensuring the reliability of the classification results.

## 2 Data acquisition

**Figure 2:**
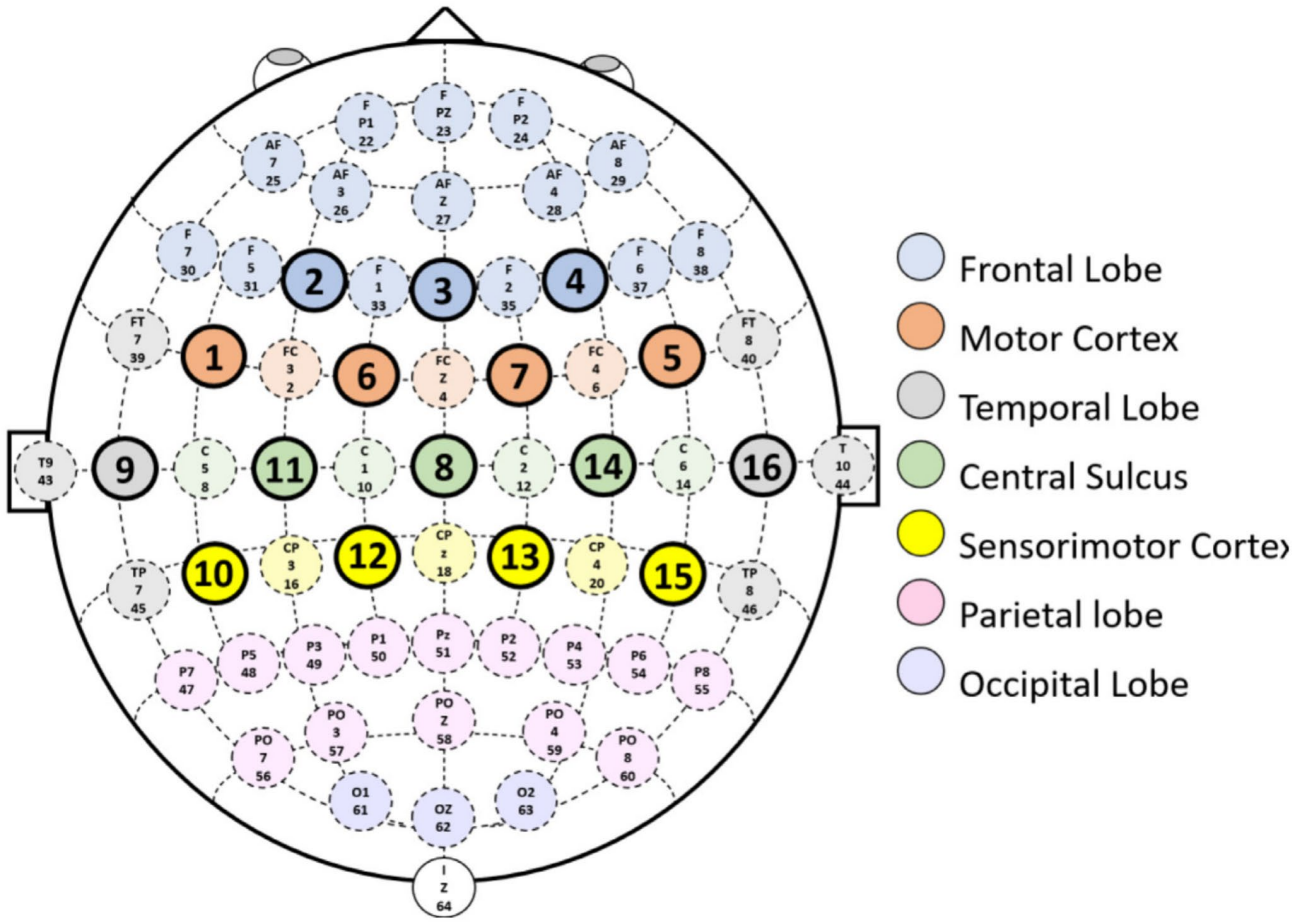
EEG electrode configuration on the 10–10 system. Reproduced from Asanza et al. (2023), licensed under CC BY 4.0.

The EEG data analyzed in this study were obtained from the publicly available MILimbEEG dataset (Asanza et al., 2023), which was selected due to its inclusion of multi-class motor execution tasks involving both upper and lower limbs, enabling a comprehensive evaluation of EEG-based brain–computer interface (BCI) systems under realistic motor control scenarios. EEG signals were acquired from 60 healthy participants using an OpenBCI Cyton + Daisy biosensing platform. Recordings were obtained from 16 dry electrodes positioned according to the international 10–10 electrode placement system, providing coverage over sensorimotor cortical regions relevant to motor execution. The spatial configuration of the electrodes and their correspondence to underlying cortical areas are illustrated in Figure 2, which highlights the distribution of channels over motor-related Brodmann areas. EEG signals were sampled at a frequency of 125 Hz. As described in the original dataset documentation, the recorded EEG signals were preprocessed using a bandpass filter between 7 and 31 Hz, preserving the *μ* (7.5–12.5 Hz) and *β* (16–31 Hz) frequency bands that are strongly associated with motor-related cortical activity, while attenuating low-frequency drift and high-frequency noise. Signal normalization was applied to reduce inter-subject variability and to standardize signal amplitudes across recording sessions. In this study, we directly utilized the preprocessed signals provided by the dataset without introducing additional filtering stages.

**Figure 3:**
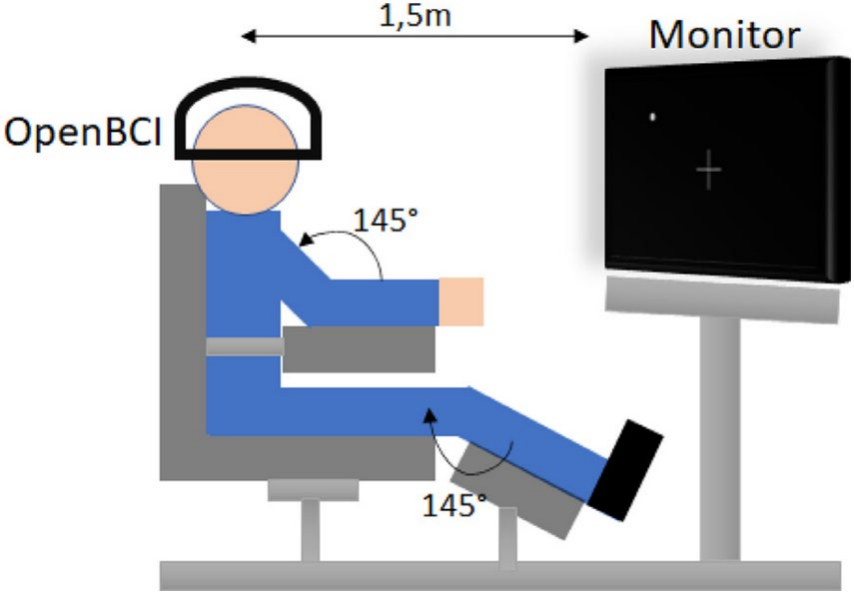
Setup of the experimental environment for EEG recording. Reproduced from Asanza et al. (2023), licensed under CC BY 4.0.

**Figure 4:**
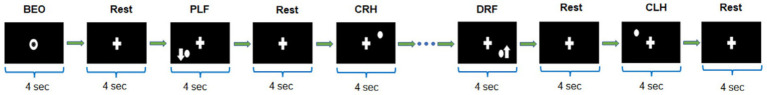
Visual timeline of motor tasks and rest intervals. Reproduced from Asanza et al. (2023), licensed under CC BY 4.0.

**Figure 5:**
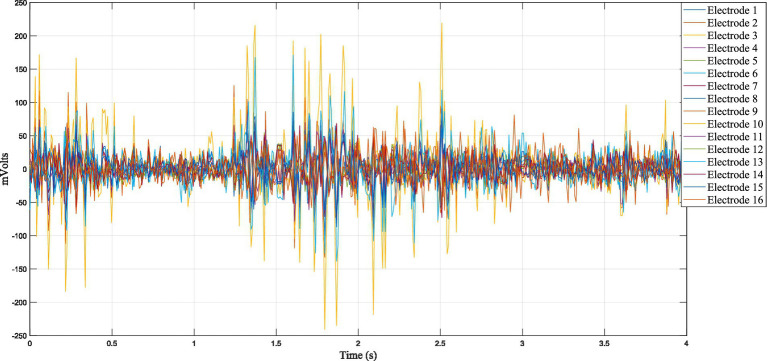
Sample EEG data trace during motor task execution.

The experimental setup adopted during data acquisition is shown in Figure 3, where participants were seated in a fixed posture to ensure consistent recording conditions across sessions. Visual cues presented on a monitor guided participants through a predefined sequence of motor tasks and rest intervals, as illustrated in Figure 4. This structured task timeline ensured temporal consistency across trials and facilitated the segmentation of EEG signals corresponding to individual motor actions. Each participant performed eight distinct motor tasks, including baseline with eyes open, left-hand closing, right-hand closing, dorsal and plantar flexion of the left and right feet, as well as resting periods between tasks. This protocol yielded a total of 480 EEG samples (60 participants × 8 tasks), with balanced representation across all task categories. A representative example of the recorded EEG signals during task execution is shown in Figure 5, illustrating the normalized EEG waveforms used for subsequent feature extraction.

Rather than reproducing the full experimental protocol detailed in the original MILimbEEG publication, this study focuses on the study-specific utilization of the dataset, including feature extraction, classification architecture, and evaluation strategy. The MILimbEEG dataset was chosen because it enables the assessment of computationally efficient learning models on a challenging eight-class motor execution problem, which extends beyond the binary or four-class motor imagery tasks commonly considered in lightweight EEG classification studies.

## 3 Feature extraction from EEG signals

Feature extraction is a pivotal process in the analysis of EEG data, particularly when these signals are used to decipher and classify brain activity related to specific tasks. Effective feature extraction transforms raw EEG data into a more manageable set of features that capture the essential characteristics of the brain signals. This transformation is crucial for enhancing the performance of machine learning models, such as neural networks, by providing them with robust and informative inputs that highlight the underlying patterns of neural activity.

In this study, we extract a comprehensive set of 10 features from the EEG signals recorded at each of 16 electrodes. These features encompass both time and frequency domain characteristics, providing a multidimensional representation of the EEG signals that are associated with various motor tasks. The extracted features include:

*Frequency band powers:* The power spectral density of the EEG signals is calculated within specific frequency bands known to be relevant to brain activity. These bands are (Han, 2025):

- Delta (0.5–4 Hz): Associated with sleep and deep relaxation.
- Theta (4–8 Hz): Linked to meditation, memory recall, and relaxation.
- Alpha (8–13 Hz): Related to states of relaxation while awake.
- Beta (13–30 Hz): Generally associated with active thinking, problem-solving, and active concentration.
- Gamma (30–50 Hz): Connected with high-level information processing and cognitive functioning.

For each band, the power is computed using the Fast Fourier Transform (FFT) of the EEG signal, and integrating the square of the FFT’s magnitude within the band limits shown in Equation 1 (Kalauzi et al., 2025):


$$P_{b}=\sum_{f=f_{\mathrm{low}}}^{f_{\text{high}}}X{\left(f\right)}^{2}$$ (1)

where *X*(*f*) is the Fast Fourier Transform (FFT) of the EEG signal, and $f_{\mathrm{low}}$ flow and $f_{\text{high}}$ are the lower and upper frequency limits of band b.

*Dominant frequency:* The dominant frequency feature pinpoints the frequency that exhibits the maximum amplitude within an EEG signal’s power spectrum, indicative of the most prominent rhythmic activity during the measured period. This feature is crucial for identifying the primary brain state associated with various cognitive or motor tasks. To determine the dominant frequency, the EEG signal is first transformed from the time domain to the frequency domain using the FFT. The power of each frequency component is then computed, and the frequency with the highest power is identified as the dominant frequency. The process can be expressed with Equations 2, 3 (Sharma et al., 2021):


$$P(f)=X{\left(f\right)}^{2}$$ (2)

$$f_{\mathrm{dom}}=\text{argmax}(P(f))$$ (3)

where *P*(*f*) represents the power at frequency *f*, and $f_{\mathrm{dom}}$ is the dominant frequency where the maximum power occurs. This feature is essential for assessing the EEG signal’s behavior and plays a pivotal role in classifying EEG data accurately for both diagnostic and interactive applications in brain-computer interfaces.

*Mean amplitude:* The average amplitude provides a measure of the signal’s average power level across the entire recording, calculated as Equation 4:


$$\mu =\frac{1}{N}\sum_{n=0}^{N-1}|x[n]|$$ (4)

where x[n] is the signal amplitude at sample *n*, and *N* is the total number of samples.

*Standard deviation of amplitude* shown in Equation 5: This statistical measure provides an index of the variability in the signal amplitude:


$$\sigma =\sqrt{\frac{1}{N}\sum_{n=0}^{N-1}{\left(|x[n]|-\mu \right)}^{2}}$$ (5)

*Median amplitude* shown in Equation 6: The median amplitude is a robust measure of the central tendency of the signal amplitude, less sensitive to outliers than the mean:


median=median(x[n]) (6)

*Peak-to-peak amplitude* shown in Equation 7: This feature measures the difference between the maximum and minimum amplitudes in the EEG signal, providing an estimate of the signal’s dynamic range (Mak et al., 2012):


ptp=max(x[n])−min(x[n]) (7)

These features were extracted for each of the 16 electrodes and for each sample, resulting in a feature matrix with dimensions corresponding to the number of samples times the product of 10 features and 16 electrodes. This comprehensive feature set serves as the input to the GMDH neural network, facilitating the precise classification of EEG signals into categories corresponding to specific motor tasks. This approach underscores the efficacy of using advanced computational models to interpret complex brain signals for applications in brain-computer interfaces and neurorehabilitation technologies.

## 4 GMDH neural network

The architecture of a GMDH network tailored for EEG signal classification involves a highly adaptive and hierarchical approach, designed to effectively model and predict complex relationships among input features that represent EEG data. The input layer of the GMDH network consists of 160 inputs. These inputs represent extracted features from EEG signals, which might include spectral powers in various frequency bands, statistical summaries of the signals (mean, ptp, etc.), and other relevant features that capture the dynamics and characteristics of the EEG during different motor tasks. Each feature provides crucial information that aids in distinguishing between the types of motor tasks. The GMDH network uses an auto-selective layer formation process where neurons in each layer are formed based on the polynomial regression models of the inputs or the outputs from the previous layers. Each neuron represents a polynomial equation (as shown in Equation 8), which might start as a simple quadratic model and can become progressively more complex in higher layers if required (Ivakhnenko, 1971):


$$y=b_{0}+b_{1}x_{1}+b_{2}x_{2}+b_{3}x_{1}^{2}+b_{4}x_{2}^{2}+b_{5}x_{1}x_{2}+\ldots$$ (8)

Here, $x_{1}.x_{2}.\ldots$ are inputs from the previous layer or the initial data layer, $b_{0}.b_{1}.b_{2}.\ldots$ are the coefficients learned during training, and y is the output used as an input for the next layer.

The architecture of the GMDH network implemented for EEG signal classification in this study is meticulously designed to optimize the process of identifying and categorizing motor-related brain activities. The network consists of a single input layer, eight hidden layers, and a concluding output layer. The input layer is tasked with handling 160 features extracted from EEG data, which encapsulate the essential characteristics necessary for initial data analysis. These features feed into a series of eight hidden layers, which are strategically structured to refine the data processing through successive stages of complexity and abstraction. The neuron count in these hidden layers is carefully configured to gradually reduce the dimensionality of the data, starting with 40 neurons in the first hidden layer and decreasing through subsequent layers to 38, 27, 22, 16, 10, 8, and finally 5 neurons in the last hidden layer. This decremental structuring aids in distilling the most salient features and interactions relevant to the motor tasks. The output layer culminates in a single neuron, which classifies the EEG data into one of the eight predefined motor task categories. This layered and progressively refining architecture ensures a balanced approach to learning, capturing deep and complex patterns in the EEG signals while avoiding overfitting, thereby enhancing the network’s ability to make accurate and robust predictions. Each class corresponds to a specific motor task and is numerically represented as follows:

(1) Baseline with Eyes Open (BEO), (2) Closing Left Hand (CLH), (3) Closing Right Hand (CRH), (4) Dorsal Flexion of Left Foot (DLF), (5) Plantar Flexion of Left Foot (PLF), (6) Dorsal Flexion of Right Foot (DRF), (7) Plantar Flexion of Right Foot (PRF), and (8) Resting between tasks (Rest).

The dataset comprised recordings from 60 participants, each performing 8 distinct motor tasks, resulting in a total of 480 samples. These samples were divided into training and testing sets, with 70% of the data randomly selected for training and the remaining 30% used for testing. The random allocation of samples ensured a balanced and representative distribution across both subsets, reducing potential biases and enhancing the robustness of the classification model. This approach facilitated a comprehensive evaluation of the model’s performance on previously unseen data, ensuring the reliability of the results.

These classifications are based on the patterns recognized in the EEG signals, allowing for the effective categorization of brain activity corresponding to each task. In MATLAB, the entire network, including its iterative development of layers and neuron connections, is coded. The training involves adjusting the polynomial coefficients to minimize classification errors on a training dataset, with validation datasets used to prevent overfitting and to optimize the network structure. The adaptability and depth of the GMDH approach make it exceptionally suitable for EEG data, which often contains subtle and complex patterns associated with different brain activities. The end-to-end training and classification implemented in MATLAB ensure that the model is both robust and accurate, capable of handling the intricacies of EEG signal classification effectively.

The reported results correspond to a subject-dependent evaluation protocol, where EEG samples from all participants are randomly divided into training and testing sets. As a result, data from the same subjects may appear in both subsets. This evaluation strategy assesses the model’s ability to discriminate motor tasks under consistent subject-specific signal characteristics. While this setup is commonly used in EEG classification studies to evaluate algorithmic performance, extending the evaluation to a subject-independent protocol (e.g., leave-one-subject-out validation) would provide deeper insight into cross-subject generalizability and is considered an important direction for future work.

## 5 Results and discussion

This study has demonstrated the effective application of a GMDH neural network in classifying EEG signals into eight distinct motor task categories with an impressive overall accuracy of 96.5% on test data. This performance highlights the network’s capability to handle complex pattern recognition tasks in neurophysiological data, a cornerstone in the development of advanced BCIs.

The accuracy achieved by the neural network is indicative of the robustness and suitability of the feature extraction techniques employed in this study. These techniques, which included analysis of spectral components, time-domain features, and statistical characteristics of EEG signals, provided a comprehensive dataset from which the network could learn. The high level of accuracy achieved suggests that the features were well-chosen, capturing essential information needed to distinguish between the motor tasks effectively. The network was carefully architected with one input layer, eight hidden layers of descending neuron counts, and one output layer. This structure allowed the network to gradually refine its understanding of the data, layer by layer, improving its predictive accuracy while avoiding the pitfalls of overfitting. This approach demonstrates the strength of using a methodical layer reduction strategy in deep learning models where the complexity of the model is balanced against its performance.

**Figure 6:**
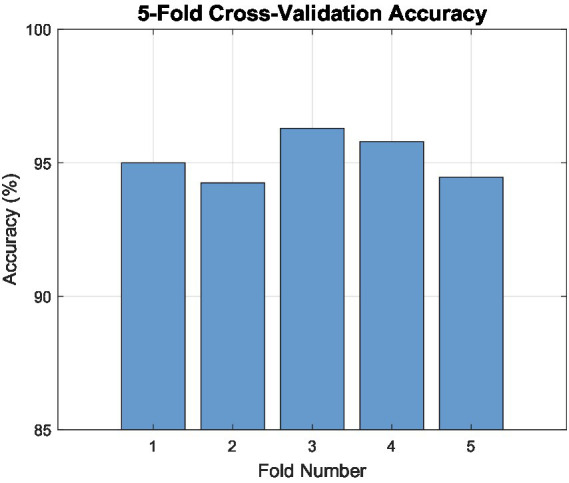
Results of 5-fold cross-validation, showing classification accuracy for each fold.

To ensure the robustness and reliability of the proposed classification approach, a 5-fold cross-validation was employed. In this method, the dataset is divided into five equally sized subsets (folds). During each iteration, four folds are used for training the model, and the remaining fold is used for testing. This process is repeated five times, ensuring that each fold is used once as the testing set. The average performance metrics from all iterations provide a comprehensive assessment of the model’s generalizability. Figure 6 illustrates the results of the 5-fold cross-validation, highlighting the classification accuracy achieved in each fold. The accuracy across folds ranged from 90 to 96%, with a mean accuracy of approximately 95%. This consistent performance across folds confirms the stability of the proposed methodology and its ability to generalize effectively across unseen data splits.

The experimental evaluation follows a two-stage protocol to ensure a fair and transparent performance assessment. First, the dataset is randomly divided into 70% training data and 30% testing data, where the test set remains completely unseen during model training and hyperparameter selection. The reported 96% classification accuracy corresponds exclusively to this held-out test set. Second, 5-fold cross-validation is applied independently on the full dataset to evaluate the stability and robustness of the proposed method across different data partitions. The cross-validation results are reported as complementary performance indicators and are not mixed with the test-set evaluation.

**Figure 7:**
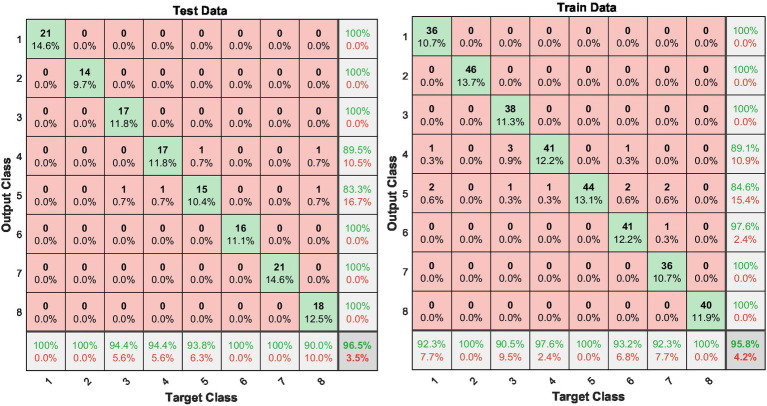
Confusion matrices for training and testing data.

The confusion matrices for both the training and testing datasets provide a clear visual representation of the model’s performance across different classes (Figure 7). For training data, high accuracy across all tasks suggests that the model has effectively learned the training patterns. However, the real test of the model’s utility is reflected in the test data, where the model must apply what it has learned to previously unseen data. Here, the model also performed exceptionally well, particularly in differentiating between more distinct tasks such as Baseline with Eyes Open (BEO) and various motor flexions. Notably, some confusion did occur between tasks that are similar in nature, as reflected in the slight mixing of classes such as Dorsal and Plantar flexions. These areas of confusion offer valuable insights into where the model might be improved through further training or enhanced feature extraction, perhaps by incorporating additional or more nuanced features that can better differentiate between similar motor activities. The confusion matrices are computed for the trained GMDH network that achieved the 96.5% test accuracy, based on the fixed train–test split. These matrices are generated separately for the training and testing phases and provide a detailed class-wise performance analysis. In particular, they illustrate the distribution of correctly classified motor tasks along the diagonal entries, as well as misclassification patterns between physiologically similar classes, such as dorsal and plantar flexion movements. This analysis enables a deeper interpretation of class-wise robustness and complements the overall accuracy and cross-validation results by revealing task-specific classification behavior.

The confusion matrix used in this study is based on a dataset comprising 480 samples, with each of the 60 subjects contributing 8 samples corresponding to distinct motor tasks. These samples were balanced across classes to ensure an unbiased evaluation of the model’s performance. To improve classification accuracy, features were extracted across multiple frequencies within the *μ* (7.5–12.5 Hz) and *β* (16–31 Hz) bands for each subject.

The computational efficiency of the proposed GMDH-based classifier was quantitatively analyzed in terms of parameter count, arithmetic operations, and inference time. The implemented architecture consists of eight hidden layers with 40–38–27–22–16–10–8–5 quadratic polynomial neurons, resulting in a total of 166 neurons. Each neuron employs a second-order polynomial with two inputs and six coefficients, yielding exactly 996 trainable parameters. During inference, each polynomial neuron requires 8 multiplications and 5 additions, leading to a total of 1,328 multiplications and 830 additions, i.e., 2,158 arithmetic operations per sample for a complete forward pass through the network. Inference time was measured using a MATLAB R2023a implementation executed in single-thread CPU mode on an Intel Core i7-12700H processor. The reported inference time corresponds to the average over 1,000 forward passes with batch size equal to one. Under these conditions, the proposed model achieved an inference time of 3.21 ms per sample. This quantitative analysis demonstrates that the proposed method combines high classification accuracy with a compact model size and fast inference, making it well suited for real-time EEG decoding and deployment on FPGA or other resource-constrained embedded platforms, where memory footprint and computational efficiency are critical.

The depth and decremental structure of the proposed GMDH network were selected to balance representational capacity and computational efficiency. Increasing network depth enables hierarchical modeling of nonlinear interactions among EEG-derived features, while progressively reducing the number of neurons across layers limits redundancy and mitigates overfitting. This architectural strategy is particularly suitable for EEG datasets with limited sample sizes, where overly complex models often fail to generalize. Rather than introducing a novel GMDH variant, this study focuses on a task-driven architectural configuration that leverages the self-organizing properties of GMDH to achieve high classification accuracy under strict computational constraints.

To provide a comprehensive assessment of the proposed method, performance evaluation was conducted using both global metrics and per-class metrics, addressing the limitations of relying solely on overall accuracy in multiclass EEG classification tasks. Table 1 summarizes the global performance metrics for both the training and testing datasets, including overall accuracy, Cohen’s kappa coefficient, and macro- and weighted-averaged precision, recall, and F1-score. On the test dataset, the proposed GMDH-based framework achieved an overall accuracy of 96.53% and a Cohen’s kappa value of 0.9602, indicating an almost perfect agreement beyond chance. The macro-averaged F1-score of 0.9650 demonstrates balanced classification performance across all classes, while the weighted F1-score of 0.9659 confirms robustness when accounting for class distribution. To further analyze class-specific behavior, Table 2 reports the per-class precision, recall, and F1-score computed on the test dataset for each motor task. This fine-grained evaluation reveals that most classes achieve perfect or near-perfect precision and recall, highlighting the strong discriminative capability of the proposed method. Slight performance degradation is observed primarily for anatomically and physiologically similar movements, such as dorsal and plantar flexion of the foot, which are known to produce overlapping EEG activation patterns. Despite this inherent challenge, the corresponding F1-scores remain high, indicating reliable and balanced classification performance.

**Table 1:** Classification performance metrics for training and testing datasets.

Dataset	Accuracy (%)	Cohen’s kappa	Macro precision	Macro recall	Macro F1	Weighted F1
Training	95.83	0.9523	0.9642	0.9574	0.9590	0.9589
Testing	96.53	0.9602	0.9660	0.9658	0.9650	0.9659

**Table 2:** Per-class classification performance on the test dataset.

Class	Motor task	Support	Precision	Recall	F1-score
1	Baseline with Eyes Open (BEO)	21	1.000	1.000	1.000
2	Closing Left Hand (CLH)	14	1.000	1.000	1.000
3	Closing Right Hand (CRH)	18	1.000	0.944	0.971
4	Dorsal Flexion of Left Foot (DLF)	18	0.895	0.944	0.919
5	Plantar Flexion of Left Foot (PLF)	16	0.833	0.938	0.882
6	Dorsal Flexion of Right Foot (DRF)	16	1.000	1.000	1.000
7	Plantar Flexion of Right Foot (PRF)	21	1.000	1.000	1.000
8	Rest	20	1.000	0.900	0.947

**Figure 8:**
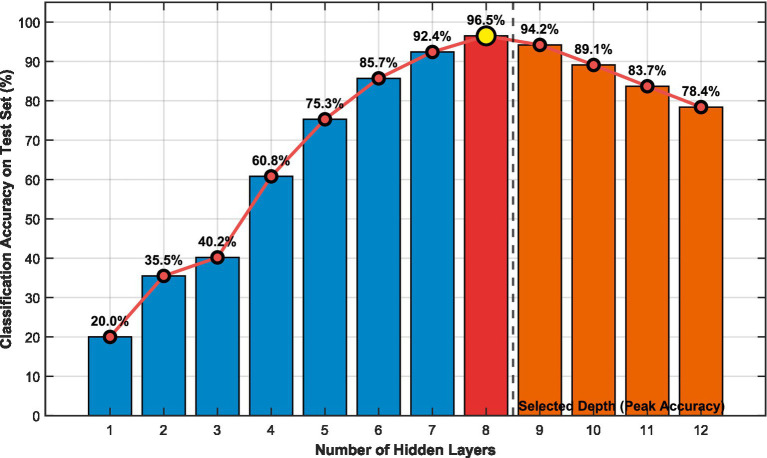
Impact of the number of hidden layers on classification accuracy in the GMDH network.

To rigorously justify the selection of 8 hidden layers in the proposed decremental GMDH architecture, we conducted an extended ablation study evaluating network depths from 1 to 12 layers (Figure 8). As illustrated, classification accuracy on the test set progressively increases with depth up to 8 layers, reaching a peak of 96.5%. This improvement reflects the benefits of hierarchical feature abstraction enabled by additional layers, allowing the self-organizing polynomial neurons to capture increasingly complex nonlinear relationships in the EEG-derived features. However, further increasing the depth beyond 8 layers results in a sharp decline in test accuracy (e.g., dropping to 94.2% at 9 layers, 89.1% at 10 layers, 83.7% at 11 layers, and 78.4% at 12 layers). This severe performance degradation is a clear indicator of overfitting, which is expected given the limited dataset size (only 480 samples). Deeper networks introduce unnecessary model complexity (higher parameter counts and inference latency) without corresponding generalization gains, as the GMDH’s self-organizing mechanism begins to fit noise rather than meaningful patterns when data is scarce. The decremental neuron arrangement (40 → 5 neurons) already mitigates redundancy, but excessive depth exacerbates overfitting risks on small datasets. Therefore, 8 layers were selected as the optimal configuration, achieving the highest accuracy.

**Figure 9:**
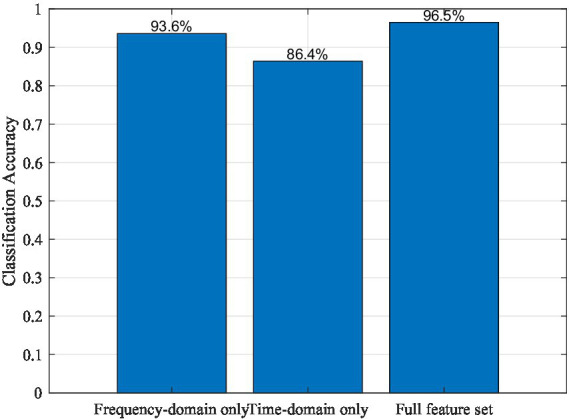
Ablation study illustrating the contribution of different EEG feature subsets to motor-task classification performance.

To further examine the rationality of the hand-crafted feature design, an ablation analysis was conducted to investigate the contribution of different feature subsets to the overall classification performance. The extracted EEG features were grouped into two main categories: frequency-domain features, including band power and dominant frequency, and time-domain statistical features, including mean, standard deviation, median, and peak-to-peak amplitude. As illustrated in Figure 9, frequency-domain features alone achieve substantially higher accuracy than time-domain features, reflecting the strong relevance of *μ* and *β* rhythms in motor-related EEG activity. However, the highest performance is obtained when both feature groups are combined, indicating that time-domain statistics provide complementary information that further enhances classification robustness.

Table 3 provides a quantitative comparison between the proposed method and representative EEG classification approaches. Unlike qualitative descriptions of computational load, this comparison reports the number of trainable parameters and inference time per sample, offering a more objective assessment of computational efficiency. While most existing methods rely on deep or hybrid architectures with automatic feature extraction and large parameter counts, the proposed GMDH-based framework achieves superior classification accuracy with three orders of magnitude fewer parameters and substantially lower inference latency. Although manual feature extraction introduces an additional preprocessing step, it is performed offline and does not affect real-time inference, making the overall framework suitable for deployment in resource-constrained and embedded BCI systems.

**Table 3:** Comparison of EEG classification methods based on accuracy, computational load, and feature extraction approach.

Study	Method	Accuracy (%)	Number of parameters	Inference time (ms/sample)	Feature extraction
(Sarma and Thamilarasu, 2024)	Deep CNN	91.2	≈1.2 × 10^6^	≈25–30	Automatic
(Flores et al., 2024)	CNN + transfer learning	89.5	≈8.5 × 10^5^	≈20–25	Automatic
(Akuthota et al., 2024)	Compact CNN	90.8	≈1.1 × 10^5^	≈8–10	Automatic
(Panthadas and Bhuiyan, 2024)	Hybrid CNN–SVM	88.7	≈9.0 × 10^5^	≈18–22	Automatic
(Devi et al., 2024)	Multimodal DNN	93.5	≈2.3 × 10^6^	≈30–40	Automatic
(Ziegelman and Hernandez, 2024)	Neural ODE	92.1	≈6.5 × 10^5^	≈15–20	Automatic
This work	GMDH neural network	96.5	996	3.21	Manual

**Figure 10:**
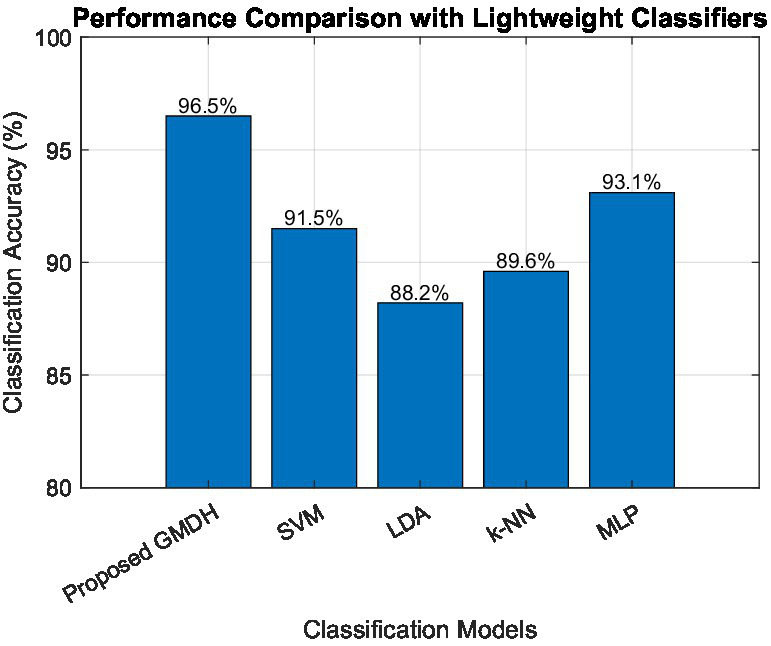
Classification accuracy comparison between the proposed GMDH model and standard lightweight classifiers.

To further assess the efficiency of the proposed approach when using handcrafted features, a comparative evaluation with several standard lightweight classifiers was performed. As shown in Figure 10, the proposed multi-layer GMDH network is compared with Support Vector Machine (SVM), Linear Discriminant Analysis (LDA), k-Nearest Neighbors (k-NN), and a lightweight Multi-Layer Perceptron (MLP). All models and networks were trained and evaluated using the same handcrafted feature set as input and under identical experimental conditions, ensuring a fair and unbiased comparison. The results indicate that the proposed GMDH model achieves the highest classification accuracy of 96.5%, outperforming the conventional classifiers.

Although Group Method of Data Handling (GMDH) is not a newly introduced learning paradigm, its application to EEG-based multi-class motor task classification remains largely underexplored, particularly in comparison with contemporary deep learning architectures. The motivation for selecting GMDH in this study is rooted in both theoretical considerations and practical constraints inherent to EEG signal analysis. From a theoretical perspective, EEG signals are characterized by strong nonlinearity, high inter-channel dependency, and limited sample availability. GMDH explicitly models nonlinear feature interactions through polynomial representations, enabling it to capture second-order and higher-order dependencies between EEG-derived features. This inductive bias is well aligned with the physiological nature of EEG, where meaningful information often arises from interactions between spatial channels and spectral components rather than isolated features. In contrast to deep neural networks such as CNNs, TCNs, or deep MLPs, which rely on large-scale data to effectively constrain their hypothesis space, GMDH employs a layer-wise self-organizing mechanism that automatically controls model complexity. Neurons and layers are selected based on external validation criteria, which inherently reduces overfitting and improves generalization in small-to-medium EEG datasets. Methodologically, the novelty of this work does not lie in modifying the original GMDH learning rule, but rather in the task-specific architectural design and integration strategy. We propose a decremental multi-layer GMDH structure tailored to EEG motor task decoding, where the gradual reduction in neuron count enforces progressive feature abstraction while maintaining computational efficiency. When combined with carefully engineered time- and frequency-domain EEG features, this design yields a robust and lightweight classification framework suitable for resource-constrained BCI systems. Therefore, the contribution of this study is positioned as a methodological framework that bridges manual EEG feature engineering with an adaptive polynomial-based learning model, offering a practical alternative to computationally intensive deep learning approaches for EEG-based BCIs.

This research not only advances the scientific understanding of EEG-based signal classification using neural networks but also holds profound practical implications, particularly in the realms of medical technology and neurorehabilitation. By achieving high accuracy in classifying motor-related EEG signals, this study lays the groundwork for significant advancements in the development of BCIs, which could revolutionize the way individuals with motor impairments interact with the world. The core achievement of this study—enabling precise interpretation of EEG signals related to specific motor tasks—paves the way for BCIs that can translate a user’s neural impulses into actual commands to operate assistive devices. Such technology can be life-changing for individuals suffering from paralysis, muscle weakness, or neurological disorders that impair their ability to perform everyday tasks. For example, someone with spinal cord injury could potentially use a BCI to control a robotic arm, wheelchair, or other assistive devices just by thinking about the movement they wish to execute.

This study addresses the critical challenge of balancing computational efficiency and classification accuracy in EEG signal processing. The proposed GMDH neural network, combined with manual feature extraction, significantly reduces computational complexity compared to deep learning approaches, enabling practical deployment on hardware platforms like FPGAs. This capability is crucial for real-time applications in resource-constrained environments, such as portable neurorehabilitation devices and assistive technologies. Extending the evaluation of the proposed framework to additional benchmark EEG datasets, such as the High Gamma Dataset (HGD) and well-established BCI Competition datasets (e.g., BCI Competition IV, Data Set 2a), is considered an important direction for future work to further assess generalizability and facilitate broader comparison with existing EEG-based motor decoding methods (Schirrmeister, 2017; Brunner et al., 2008).

Despite these strengths, several limitations warrant discussion. First, the reliance on manual feature extraction requires domain expertise and may limit the generalizability of the approach to datasets with varying signal characteristics. Second, while the model achieves state-of-the-art accuracy, its performance has been evaluated primarily on a specific dataset, necessitating further validation on diverse datasets to confirm its robustness. Lastly, the scalability of the proposed methodology for large-scale EEG data remains an area for future investigation.

## 6 Conclusion

This research successfully implemented a GMDH neural network to classify EEG signals into eight distinct motor tasks, achieving an impressive accuracy of approximately 96%. The meticulously designed network architecture, featuring one input layer, eight hidden layers with progressively decreasing neuron counts, and a single output neuron, effectively managed the complex patterns inherent in EEG data. The high classification accuracy highlights the effectiveness of the feature extraction techniques employed, which captured essential characteristics of EEG signals related to various motor functions. The robustness of the model was underscored by the confusion matrices for both training and testing phases, which demonstrated high accuracy across most tasks, showing promising potential for real-world applications.

The practical implications of this study are significant, especially in the development of BCIs that can dramatically improve the quality of life for individuals with motor impairments. Future work will aim to refine the EEG feature set and network architecture, expand the dataset to improve the model’s generalizability, and explore real-time applications in neurorehabilitation and consumer electronics. As research progresses, integrating these findings into commercially viable solutions will necessitate thorough testing and validation through clinical trials to ensure they meet the practical needs of users while adhering to medical safety standards. The continued exploration at the intersection of AI, machine learning, and neuroscience holds the promise of expanding the boundaries of medical science and enhancing patient care, marking a step toward a future where technology and human health care converge to create more inclusive, effective, and personalized therapies.

## Data Availability

Publicly available datasets were analyzed in this study. This data can be found at: https://data.mendeley.com/datasets/x8psbz3f6x/2.
